# Background mutation in strain RB2126 affects the locomotion behavior of *flp-1* mutants

**DOI:** 10.17912/micropub.biology.000393

**Published:** 2021-05-09

**Authors:** Irini Topalidou

**Affiliations:** 1 Department of Biochemistry, University of Washington, Seattle, WA 98195

## Abstract

The FLP-1 neuropeptide is involved in locomotion, mechanosensation, and reproduction. Strain RB2126 is reported to contain the *flp-1(ok2811)* mutation, a prospective null allele. Here we report that strain RB2126, in addition to *flp-1(ok2811)*, also contains two additional background mutations. One of the mutations gives the animals a constitutive dauer phenotype (Daf-c), and complementation testing confirms that it is in the Daf-c gene *daf-11. *The second mutation is isolated based on the fact that it partially suppresses the elevated body curvature of *flp-1(ok2811)* mutants, but it remains uncloned. The updated genotype of RB2126 strain is* flp-1(ok2811); daf-11(yak155); yak156.*

**Figure 1. Strain RB2126 contains background mutation(s) that affect the locomotion behavior of flp-1(ok2811) mutants:**
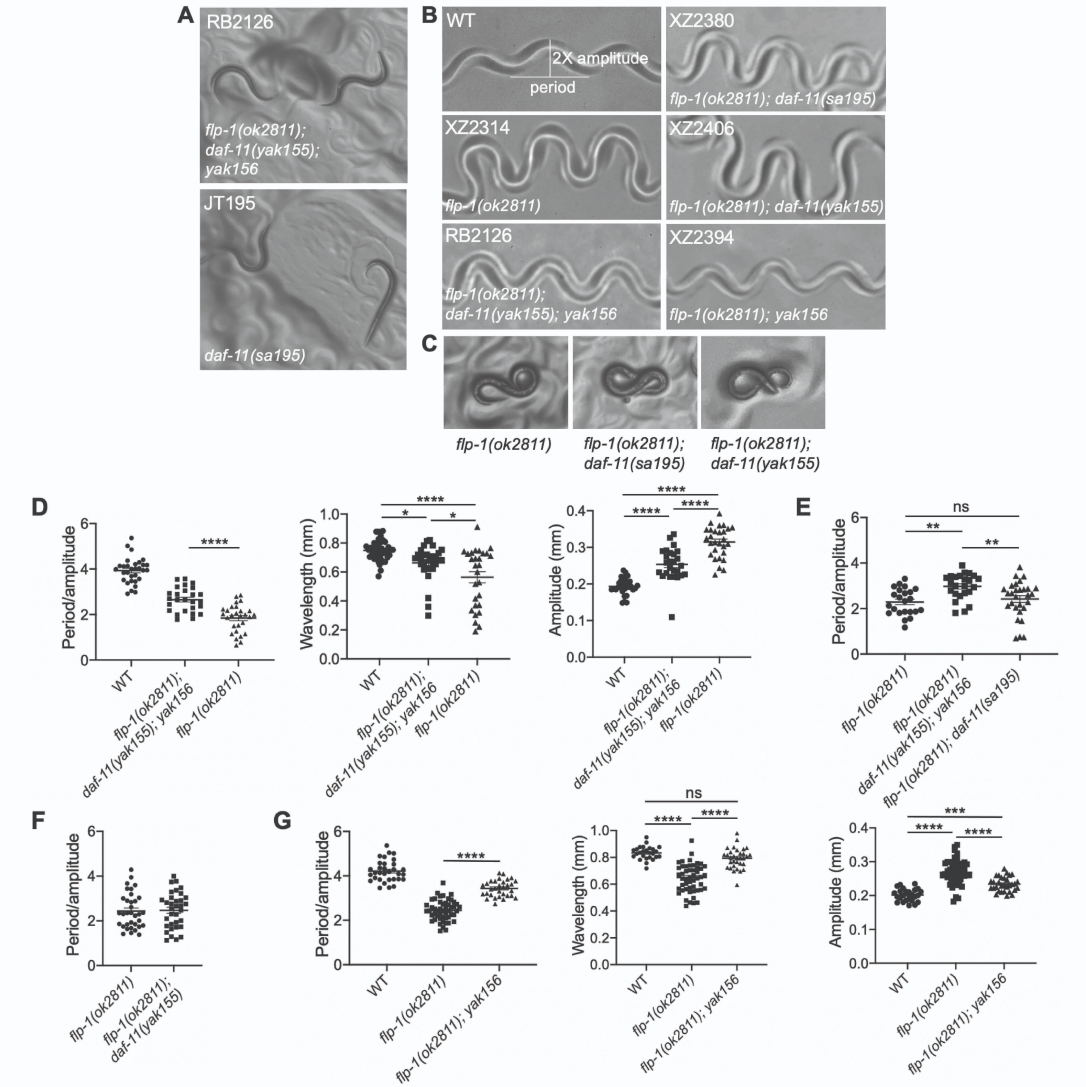
(A) Strain RB2126 (*flp-1(ok2811); daf-11(yak155); yak156)* contains a background mutation in a constitutive dauer gene. Representative photos of dauers from the RB2126 strain (Upper panel) and the *daf-11(sa195)* mutant strain JT195 (Lower panel) grown at room temperature (23˚C). (B) Strain RB2126 has background mutation(s) that reduce the deep body bends of *flp-1(ok2811*) mutants. Photos of worm tracks from wild type (WT), XZ2314 *flp-1(ok2811)*, RB2126 *flp-1(ok2811); daf-11(yak155); yak156,* XZ2380 *flp-1(ok2811); daf-11(sa195)*, XZ2406 *flp-1(ok2811); daf-11(yak155)*, and XZ2394 *flp-1(ok2811); yak156* strains. (C) Representative photos of young (~L3) XZ2314 *flp-1(ok2811)*, XZ2380 *flp-1(ok2811); daf-11(sa195)*, and XZ406 *flp-1(ok2811); daf-11(yak155)* strains showing the characteristic ”8”-like body posture. (D), (E), (F), (G) Quantification of the track waveform of the strains shown in Figure 1B (see Methods). The wavelength and amplitude of sinusoidal tracks in millimeters (mm) of strains N2 (WT), XZ2314 *flp-1(ok2811)*, RB2126 *flp-1(ok2811); daf-11(yak155); yak156,* and XZ2394 *flp-1(ok2811); yak156* are also shown in figures (D) and (G). *, P<0.05; **, P<0.01; ***, P<0.001; ****, P<0.0001; ns, not significant. Error bars = SEM; n= 24-45.

## Description

The FLP family of neuropeptides contains at least 30 genes in *C*. *elegans* (Li and Kim, 2008). Some of the *flp* genes were found to play roles in locomotion, egg laying, and sleep, but most *flp* genes have unknown function (Li and Kim, 2008; Chang *et al.* 2015). *flp-1* is one of the few *flp* genes whose mutations are reported to cause overt behavioral defects (Nelson *et al.* 1998; Hums *et al.* 2016; Buntschuh *et al.* 2018; Oranth *et al.* 2018). The initial *flp-1* deletion alleles that were analyzed behaviorally (*yn2* and *yn4*; Nelson *et al.* 1998) included a deletion of part of *daf-10* (Ailion and Thomas, 2003; Buntschuh *et al.* 2018). This resulted in some of the initial behaviors that were attributed to FLP-1 peptides being caused by loss of *daf-10* activity (Buntschuh *et al.* 2018). Subsequent analysis of newer *flp-1* deletion alleles suggests that FLP-1 peptides are involved in nose touch sensitivity, locomotion, and reproduction (Buntschuh *et al.* 2018).

*flp-1*(*ok2811)* is a prospective null allele (Hums **et al.*,* 2016; Buntschuh *et al.*. 2018). We obtained strain RB2126 (Caenorhabditis Genetics Center, CGC) in order to analyze *flp-1(ok2811)* null mutant locomotion behavior. When the strain was grown at room temperature (23°C), some animals formed noticeable dauers similar to strains containing mutations in constitutive dauer (Daf-c) genes (Hu, 2007) (Fig. 1A). To examine whether the Daf-c phenotype was because of a background mutation, strain RB2126 was outcrossed three times. This outcross resulted in the segregation of two different mutants with readily apparent phenotypes: one resulting in deep body bends and a second in the Daf-c phenotype. PCR genotyping showed that the deep body bends co-segregated with the *flp-1(ok2811)* deletion. The mutation causing the Daf-c phenotype was mapped to chromosome V close to the *daf-11* gene and a complementation test using *daf-11(sa195)* (a presumed null allele of *daf-11*) showed that it is in *daf-11* (Birnby *et al.* 2000). These results suggest that *flp-1(ok2811)* mutants exhibit elevated body curvature, and that strain RB2126 has a background mutation in *daf-11*. This new *daf-11* allele in strain RB2126was named *yak155*.

*flp-1(ok2811)* mutants are known to exhibit elevated body curvature (Nelson *et al.* 1998; Chang *et al.* 2015; Hums *et al.* 2016, Buntschuh *et al.* 2018). Interestingly, strain RB2126 (*flp-1(ok2811); daf-11(yak155)*) had significantly reduced deep body bends in comparison to strain XZ2314 (*flp-1(ok2811*); three times outcrossed from strain RB2126) (Fig. 1B, 1D). This suggests that either the *daf-11(yak155)* mutation partially suppresses the phenotype of *flp-1(ok2811)* or that the RB2126 strain has other background mutation(s) that affect the body curvature*.* To test the possibility that the reduced deep body bends of *flp-1(ok2811)* in RB2126 strain are due to *daf-11*, double mutant strain *flp-1(ok2811); daf-11(sa195)* was constructed, and its body curvature was examined. *flp-1(ok2811); daf-11(sa195)* mutants (strain XZ2380) had similar body curvature as *flp-1(ok2811)* mutants (strain XZ2314) (Fig. 1B-1E). In addition, larvae (L2 to L3 stage) of *flp-1(ok2811)* and *flp-1(ok2811); daf-11(sa195)* often exhibit a characteristic body posture resembling the number ”8”, but this posture was not observed in RB2126 larvae (Fig. 1C). This result shows that *daf-11(sa195)* does not affect the elevated body curvature of *flp-1(ok2811)* mutants, suggesting that the body curvature of the RB2126 strain may be modified by another background mutation(s) other than *daf-11(yak155)*.

To verify the presence of additional background mutation(s), the body curvature of *flp-1(ok2811); daf-11(yak155)* double mutants (strain XZ2406) and *flp-1(ok2811)* mutants (strain XZ2314) were compared. Body curvature in these strains was found to be similar, suggesting that strain RB2126 has at least one additional background mutation in an unknown gene that affects the body curvature of *flp-1(ok2811)* (Fig. 1C, 1F)*.* In support of this, strain RB2126 was outcrossed using strain EG7765 that contains the genetic marker *oxTi76* (*oxTi76* contains a transposon insertion of *Peft-3::GFP::H2B::tbb-2 3’UTR* on chromosome VI, in a location close to the *flp-1* gene) and animals of the F2 generation that were not green, and did not exhibit Daf-c or elevated body curvature were picked. This outcross resulted in the isolation of strain XZ2394 that contains the *flp-1(ok2811)* mutation but does not show the same extent of elevated body curvature as XZ2314(*flp-1(ok2811)*) strain (Fig. 1G). This suggests that strain RB2126, besides the mutation in *daf-11,* contains an additional mutation that partially but significantly suppresses the *flp-1(ok2811)* elevated body posture. This new allelewas named *yak156* and the updated genotype of strain RB2126 is *flp-1(ok2811); daf-11(yak155); yak156.*

## Methods

*Strain maintenance*

Strains were maintained at 15˚C or room temperature (23˚C) on NGM plates with *E. coli* strain OP50 as a food source (Brenner, 1974).

*Waveform quantification*

First-day adult animals were placed on an OP50 plate and allowed to move forward until they had completed at least five tracks. Each animal’s tracks were imaged at 50X magnification using a Nikon SMZ745 stereoscope and an iLabCam. Worm track period and 2X amplitude were measured using the line tool in Image J. For each worm, three to five periods and amplitudes were measured. Each experiment was performed on 5-10 worms.

*Statistics*

P values were determined using GraphPad Prism 9.0 (GraphPad Software). Normally distributed data sets requiring multiple comparisons were analyzed by a one-way ANOVA followed by a Bonferroni test. Normally distributed pairwise data comparisons were analyzed by two-tailed unpaired t tests.

*Strains*

Bristol N2: wild type

EG7765: *oxTi76[Peft-3::GFP::H2B::tbb-2 3’UTR, unc-18(+)] IV; unc-18(md299) X*

JT195: *daf-11(sa195) V*

JT6709: *unc-42(e270) V daf-11(sa195) V*

RB2126: *flp-1(ok2811) IV; daf-11(yak155) V; yak156*

XZ2314: *flp-1(ok2811)* *IV* [3X outcrossed from RB2126]

ΧΖ2370: *oxTi76[Peft-3::GFP::H2B::tbb-2 3’UTR, unc-18(+)] IV; daf-11(yak155) V*

XZ2380: *flp-1(ok2811) IV; daf-11(sa195) V*

XZ2394: *flp-1(ok2811) IV; yak156*

XZ2406: *flp-1(ok2811) IV; daf-11(yak155) V*
